# The Survival of the Unfit in Human Dentition

**Published:** 1889-07

**Authors:** J. C. Parker


### The Survival of the Unfit in Human Dentition.

DR. J. C. PARKER.

Read before the State Dental Society, June 6th, 1889.

It is probably true that in every broad generalization there is an element of untruth, or at least, the truth as stated is liable to serious misconstruction, and in no case is this more signally manifested than in the phrase of Herbert Spencer, "The survival of the fittest."

Because in its broadest sense the fittest does survive, many have jumped to the conclusion that all that "survives" is the fittest. Fitness may be defined as right relations with environment, and when it becomes apparent that just in proportion as that which survives does not sustain the best conceivable relations with its environment, does not subserve the best possible use, or is faulty in material, form, or growth, just in that proportion the fittest has not survived.

It is doubtful if there is in the human organization any more striking example of the "survival of the unfit" than in human dentition. It seems to me that it goes without saying, that given the unlimited resources of nature, the dentist who could not devise a better apparatus to conserve and promote the growth, nourishment, comfort, and welfare of the human machine than that presented to him every day in the form and material of the human teeth, would be a bungler, indeed!

No matter along what line of development the teeth may have come, there can be no question that, so far as subserving the best interests of that organization that to-day stands as the type of the highest in the universe, they are an unmitigated failure; and the presence here to-day of those I see around me, many of them grown old and bald-headed in their endeavors to patch up nature's blunders, emphasizes more distinctly than any words of mine can the stern fact of this survival of the unfit.

Let us consider first the unfitness of material that enters into the human teeth; more especially the dentine, composed as it is of an easily separated basic acid, and combined with one of the most readily decomposable of all organic tissues, gelatine, the wonder is that it so long resists the chemical elements that are always present to carry on the work of destruction.

It is true that in the substance and construction of the best varieties of enamel we have one of the most enduring of organic tissues, but its presence in such limited quantities only makes the more apparent the faultiness of all the rest.

Wouldn't you, if you had been constructing a masticating machine, have put into it the best materials you had in the shop, and in the most perfect manner? Wouldn't you have made a tooth of solid enamel from cusp to fang? And can you conceive of any state of mind—outside of lunacy—that would induce you, from a constructive point of view, to wrap up in an unyielding cell such an unmitigated bit of natural cussedness as the "dental pulp?" A modicum of the most sensitive tissue in the whole body, serving—so far as human knowledge goes—no useful or benificent purpose, placed in a position where it is subject to all the accidents of use and decay and deprived of the powers of repair when wounded, that is granted to nearly all the other soft tissues of the body, and when dead becoming the ever-present night-mare of our profession.

Did any of you ever stop and think how well the world might wag along without us if it were not for this little bit of unsanctified and unregenerated flesh? With no better material or more perfect construction than we have now, if we could eliminate this patent survival of unfitness a thousand ills that plague us now would have no existence.

You may think if we had no warning of decay, made manifest by the pain induced by sensitive dentine, we would never heed it; but experience would be the same teacher then as now, and if we found that our health and comfort was enhanced by the care of, and filling decaying teeth, don't you think we would have it done just as readily then as now? Especially if it wouldn't hurt!

Let us also consider the growth and eruption of the teeth. Has any one ever discovered any intelligent use or excuse for the deciduous teeth? Why not deciduous fingers, ears, and noses as well? Who would ever construct a masticating machine in such a blundering way? A few small insignificant teeth causing oftentimes great distress, and even death in their formation and eruption through the gums, and when once in place subject to sudden and rapid decay with all its attendant evils.

And when at length absorption occurs and the permanent teeth take their place, we often find that nature has blundered again in not making the jaw large enough, and we have presented to us those frightful irregularities that are the agony of patient and friend, and the despair of the dentist.

The elaboration of this subject might be carried on through the whole range of our professional experiences, for is not all we attempt an effort on our part to render more fit that which, through a bias in development is found unfit for the use and comfort of the human machine.

Office Boy (to editor)—" There's a mad old gent outside, sir, wat wants to see you."

Editor—" Did he say what he wanted?"

Office Boy—" Yes; sir; he said that you printed a poem that his son writ, an' he says he'll have satisfaction or go to a hospital."—*Once a Week.*

